# The Molecular Mechanisms of Tanshinone IIA on the Apoptosis and Arrest of Human Esophageal Carcinoma Cells

**DOI:** 10.1155/2014/582730

**Published:** 2014-04-15

**Authors:** Jiang-Feng Wang, Jian-Guo Feng, Jing Han, Bei-Bei Zhang, Wei-Min Mao

**Affiliations:** ^1^Cancer Research Institute, Zhejiang Cancer Hospital, No. 38 Guangji Road, Hangzhou, Zhejiang 310022, China; ^2^Key Laboratory Diagnosis and Treatment Technology on Thoracic Oncology, Hangzhou, Zhejiang 310022, China

## Abstract

*Objective.* To explore the possible mechanisms of Tanshinone IIA (TanIIA) on esophageal carcinoma cell lines. * Methods.* Two human esophageal carcinoma cell lines (EC-1 cells and ECa-109 cells) were treated with different concentrations of TanIIA. Cell proliferation was measured by CCK-8, colony-forming efficiency was calculated, cell cycle and apoptosis were measured, and changes in cell cycle- and apoptosis-related gene expression were measured by Western blotting. * Results.* The CCK-8 and colony formation assay indicated that TanIIA inhibited the cell proliferation of human esophageal cancer cells (IC50 below 1 **μ**g/mL) at 48 h. Hoechst 33258 and flow cytometry showed that TanIIA induced apoptosis in both esophageal cancer cell lines. Flow cytometry showed that TanIIA arrested cell cycle in S phase and G2/M phase. Western blotting analysis showed that Akt1 and its phosphorylation were inhibited, the Bax/Bcl-2 ratio increased, and both caspase-9 and caspase-3 were activated after treatment with 1.3 **μ**g/mL TanIIA at 48 h. Meanwhile, p53 and p21 protein levels increased, whereas cyclin B1, CDC2, and CDC2 phosphorylation were inhibited. * Conclusion.* TanIIA inhibits the growth of esophageal cancer cells and induces apoptosis in a time-dependent and concentration-dependent manner, possibly by affecting cell cycle- and apoptosis-related signaling pathways.

## 1. Introduction

Esophageal cancer is the sixth leading cause of cancer-related death in the world [1]. The north-central region of China has the highest incidence of esophageal cancer in China; 90% of these cases are squamous cell carcinomas [2]. Although the development of treatments (surgery, chemotherapy, and/or radiotherapy) for esophageal cancer has been rapid, the prognosis for patients with advanced cancer has not improved. At the same time, side effects and multidrug resistance have restricted the dose of chemotherapy drugs and radiation [3, 4]. Therefore, identification of a treatment with fewer side effects for esophageal cancer will be helpful in clinical applications or as an adjunctive reagent.

**Figure 1:**
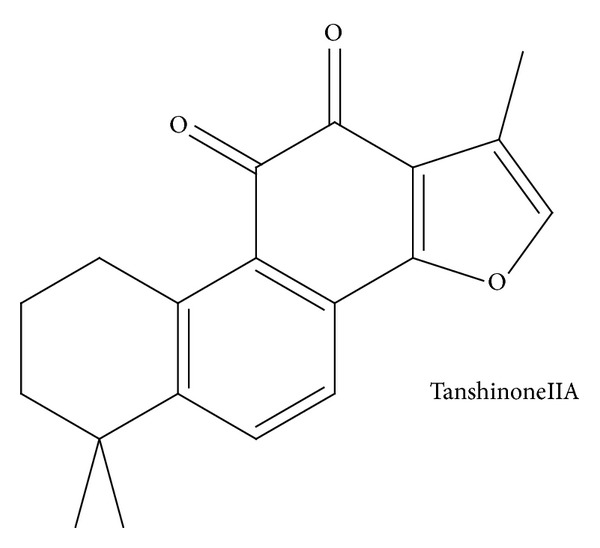
Chemical structure of Tanshinone IIA (TanIIA).

Tanshinone IIA (TanIIA, C_19_H_18_O_3_; Figure 1) is a traditional Chinese medicine, extracted from* Salvia miltiorrhiza* Bunge (Danshen), used for the treatment of cardiovascular disease in the clinic. TanIIA can protect cardiomyocytes against oxidative stress and inflammation and is widely used for coronary heart disease, angina pectoris, and other cardiovascular diseases [5, 6]. Recently, more and more research on TanIIA has focused on its anticancer effects. The evidence currently suggests that TanIIA is an anticancer agent in a variety of tumor cells, including leukemia, breast cancer, gastric cancer, and liver cancer [7–9]. Although its anticancer effect has been observed in many tumor cells, the effect of TanIIA on esophageal cancer and its molecular mechanism have not been documented. Therefore, in the present study, we evaluated the effect and molecular mechanisms of TanIIA on esophageal cancer cells (EC-1 and ECa-109 cells)* in vitro*.

## 2. Materials and Methods

### 2.1. Chemicals and Reagents

TanIIA (purity > 98%, NM_20100205) was purchased from the Zhejiang Institute of Drug Control (Zhejiang, China). RPMI-1640, fetal bovine serum (FBS), penicillin, pancreatic enzyme, and streptomycin were purchased from Hyclone (Logan, USA). All the rabbit polyclonal antibodies and HRP-conjugated secondary antibodies were purchased from Abcam (California, USA).

### 2.2. Cell Lines and Culture

The human esophageal carcinoma cell lines EC-1 and ECa-109 were generously provided from the First Affiliated Hospital of Zhengzhou University. Cells were maintained in flasks in RPMI-1640 with 10% fetal bovine serum (FBS), 100 *μ*g/mL penicillin, and 100 *μ*g/mL streptomycin, at 37°C in a humidified atmosphere of 5% CO_2_.

### 2.3. Cell Proliferation Assay

5 × 10^3^ cells/well of EC-1 and ECa-109 cells were seeded in 96-well plates for 24 h before exposure to drug treatment. Tanshinone IIA was added at various concentrations (0.3, 0.6, 1.3, 2.5, and 5.0 *μ*g/mL), with five wells used for each concentration. After treatment with Tanshinone IIA for 24, 48, and 72 h, 10 *μ*L of Cell Counting Kit 8 (CCK-8) reagents (Dojindo Laboratories, Japan) was added to each well and incubated at 37°C for 2.5 h. Absorbance was measured at 450 nm in a spectrophotometer (Thermo, USA). Each experiment was repeated three times.

### 2.4. Colony Formation Assay

3 × 10^3^ cells/well of EC-1 and ECa-109 cells were seeded in 6-well plates for 24 h before exposure to drug treatment. Tanshinone IIA was added at various concentrations (0.6 and 1.3 *μ*g/mL), with three wells used for each concentration. After treatment with Tanshinone IIA for 48 h, the treatment medium was discarded and the cells were washed with 1 × PBS and exchanged with medium containing 10% FBS. All the cells were cultured in a humidified CO_2_ atmosphere at 37°C for 5 days. The medium was decanted and each well was washed twice with 1 × PBS. The cells were stained with Giemsa (in 75% ethanol) for 10 min. Colonies (>50 cells/colony) were counted.

### 2.5. Cell Apoptosis Assay

The cell apoptosis was determined with Hoechst 33258 after EC-1 and ECa-109 cells were treated with 0.6 and 1.3 *μ*g/mL Tanshinone IIA for 48 h, respectively. After 48 h, cells were washed with 1 × PBS three times and stained with 1 *μ*g/mL of Hoechst 33258 nuclear dye (Beyotime, China) for 30 min at 37°C. Images were obtained by fluorescence microscopy (Nikon Ti-S, Japan).

### 2.6. Flow Cytometry Analysis

Flow cytometry analysis was used to determine the distribution of cells in the cell cycle and apoptosis. Briefly, EC-1 and ECa-109 cells were treated with 0.6 and 1.3 *μ*g/mL of Tanshinone IIA for 48 h. After the 48 h incubation, cells were collected by trypsinization, washed twice with ice-cold 1 × PBS, suspended in 70% alcohol, and kept at 4°C overnight. Cells were stained with the Cycle Test Plus (Becton Dickinson, USA) and Annexin V-FITC/PI (Signalway Antibody, USA). The cell cycle distribution and apoptotic cells were detected with FCM (BD FACSCalibur, USA) and analyzed with Cell Quest software (Becton Dickinson, USA).

### 2.7. Western Blotting Analysis

Total protein was extracted with RIPA and PMSF buffer and quantified using a bicinchoninic acid (BCA) protein assay kit (Beyotime, China). A total of 40 *μ*g of protein was subjected to 12% SDS-PAGE electrophoresis. After SDS-PAGE, proteins were transferred to a polyvinylidene difluoride (PVDF) membrane. The membrane was blocked with 5% skim milk powder (OXOID, UK) for 2 h and then incubated with primary antibody at 4°C overnight. After three washes with 1 × TBST, the membrane was incubated with secondary antibodies (HRP-conjugated 1 : 1000) at room temperature for 2 h. The intensity of the specific immunoreactive bands was detected by enhanced chemiluminescence (ECL) after washing with 1 × TBST three times again. Antibodies for Akt1 (ab32505; 1 : 500), phospho-Akt1 (pS473, ab81283; 1 : 500), p53 (ab32389; 1 : 500), Bax (ab32505; 1 : 1,000), Bcl-2 (ab32124; 1 : 250), procaspase-9 (ab32539; 1 : 500), cleaved-caspase-9 (ab133520; 1 : 500), caspase-3 (ab32351; 1 : 500), caspase-3 active (ab32042; 1 : 500), p21 (ab109520; 1 : 500), CDC2 (ab133327; 1 : 1000), phospho-CDC2 (Py15, ab133463, 1 : 1000), cyclin B1 (ab32053; 1 : 1000), and beta-tubulin (ab108342; 1 : 1000) were from Abcam.

### 2.8. Statistical Analysis

Data are presented as the means ± SD. To examine differences between the groups one-way analysis of variance (ANOVA) was performed using SPSS 13 software (SPSS Inc., Chicago, IL, USA). *P* < 0.05 was considered to indicate a statistically significant difference.

## 3. Results

### 3.1. TanIIA Arrest the Transaction of G2 Phase to M Phase to Inhibit the Proliferation of Esophageal Carcinoma Cells

**Figure 2:**
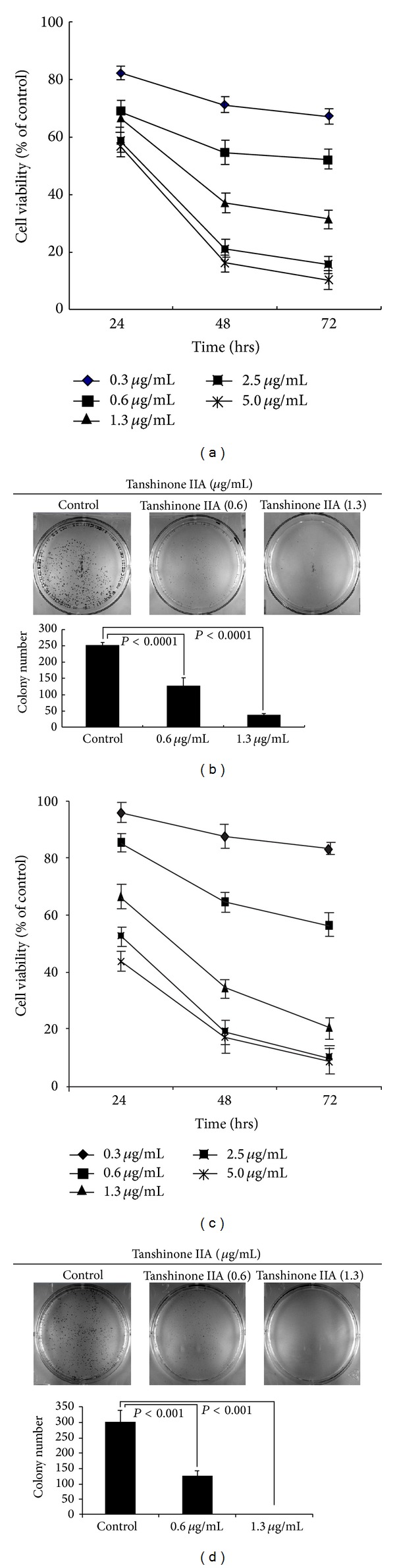
TanIIA inhibits the growth of esophageal cancer cells in a time- and concentration-dependent manner. (a) EC-1 cell; (c) ECa-109 cell. Cells were seeded onto 96-well plate at 5 × 10^3^ cells/well and were treated with increasing concentrations of 0.3, 0.6, 1.3, 2.5, and 5.0 *μ*g/mL TanIIA for 24, 48 h, and 72 h, respectively. Cell viability was determined by the CCK-8 assay. Results are performed by independent experiments in triplicate. (b) EC-1 cell; (d) ECa-109 cell. Cells were seeded onto 6-well plate at 3000/well and were treated with concentrations of 0.6 and 1.3 *μ*g/mL TanIIA for 48 h and maintained for 5 days, respectively. Colony formations were fixed by methanol and acetic acid (3 : 1) and stained with Giemsa. Data represent means ± SD. *P < 0.001 versus control.

We exposed EC-1 and ECa-109 cells to different concentrations (0.3, 0.6, 1.3, 2.5, and 5.0 *μ*g/mL) of TanIIA for 24 h, 48 h, and 72 h. Cell viability was observed to be time-dependent and concentration-dependent (*P* < 0.05) (Figures 2(a) and 2(c)). From the results of the CCK-8 assay, we chose to apply 0.6 and 1.3 *μ*g/mL TanIIA to EC-1 and ECa-109 cells for 48 h. Colony formation also decreased in a concentration-dependent manner (Figures 2(b) and 2(d)). The IC50 of TanIIA at 48 h was 0.75 *μ*g/mL in EC-1 cells and 0.93 *μ*g/mL in ECa-109 cells, respectively. We used flow cytometry to assess the cell cycle status after treatment with TanIIA (0.6 and 1.3 *μ*g/mL) on EC-1 and ECa-109 cells for 48 h. TanIIA induced cell cycle arrest at the S and G2/M phases and increased the number of cells in the S and G2/M phases in EC-1 and ECa-109 cells lines. Treatment of EC-1 and ECa-109 cells with 1.3 *μ*g/mL TanIIA at 48 h significantly increased the percentage of cells in S phase [(46.44 ± 2.26)% and (40.84 ± 6.41)%, resp.] compared with the control group [(28.55 ± 0.34)% and (33.89 ± 1.98)%; *P* < 0.05] (Figure 5). The percentage of cells in G2/M phase also increased [(14.11 ± 1.56)% and (15.16 ± 3.44)%] compared with the control group [(9.51 ± 2.03)% and (8.99 ± 1.62)%; *P* < 0.05]. Treatment of EC-1 and ECa-109 cells with 0.6 *μ*g/mL TanIIA caused no obvious increases at 48 h.

### 3.2. TanIIA Induces Apoptosis in Esophageal Carcinoma Cells

**Figure 3:**
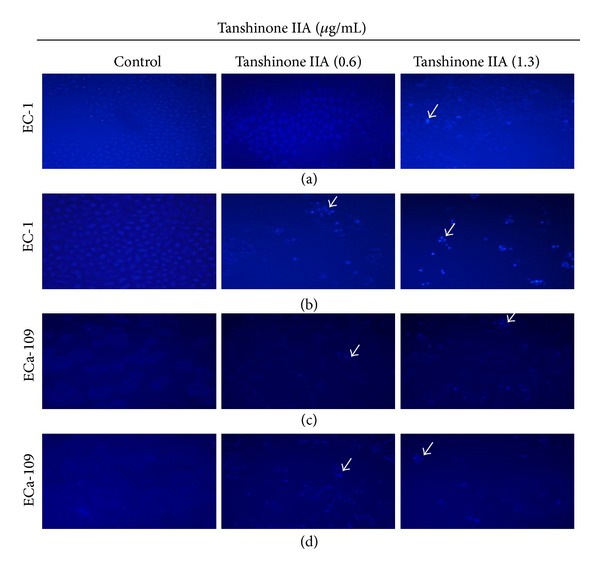
TanIIA induces esophageal cancer cells apoptosis at 24 h and 48 h. ((a), (b)) EC-1 cell: (a) at 24 h; (b) at 48 h; ((c), (d)) ECa-109 cell: (c) at 24 h; (d) at 48 h. Nuclear morphology of cells stained with Hoechst-33258 was analyzed by fluorescence microscopy (×100) at 24 and 48 h by concentrations of 0.6 and 1.3 *μ*g/mL TanIIA. Apoptotic bodies were pointed out by white arrows.

**Figure 4:**
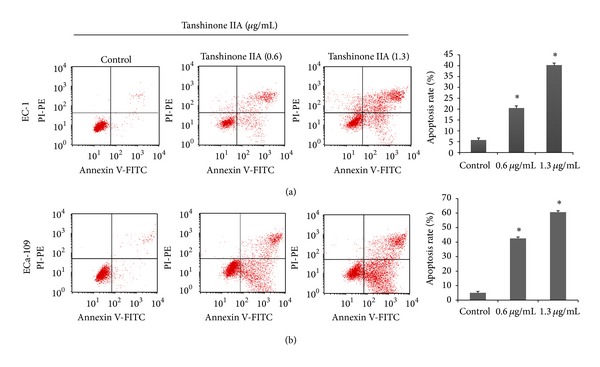
Rate of apoptosis induced by TanIIA in esophageal cancer cells at 48 h. (a) EC-1 cell; (b) ECa-109 cell. Apoptosis was analyzed by Annexin V-FITC/PI staining at 48 h by concentrations of 0.6 and 1.3 *μ*g/mL TanIIA. Data represent means ± SD. *P < 0.05 versus control.

**Figure 5:**
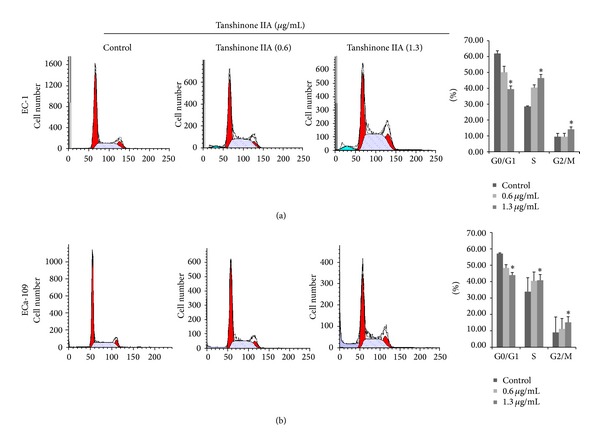
Cell cycle analysis of esophageal cancer cells treated with TanIIA. (a) EC-1 cell; (b) ECa-109 cell. Cells were treated with concentrations of 0.6 and 1.3 *μ*g/mL TanIIA for 48 h, respectively. Both the control and treated cells were harvested and subjected to flow cytometric analysis. After TanIIA treatment, cells in S and G2/M phase increased, and the cells in G0/G1 phase decreased. Data represent means ± SD. *P < 0.05 versus control.

Two methods were used to assess apoptosis after TanIIA treatment (0.6 and 1.3 *μ*g/mL) on EC-1 and ECa-109 cells for 24 h and 48 h: Hoechst and Annexin V-FITC/PI staining. In Hoechst 33258 staining, the nuclear morphology of EC-1 and ECa-109 cells in the control group was normal (Figure 3). There were relatively few changes 24 h after treatment with TanIIA. In contrast, 48 h after treatment with TanIIA, significant nuclear condensation and morphological changes, such as chromatin condensation and fragmentation, were observed in both EC-1 and ECa-109 cells. In Annexin V-FITC/PI double staining by FACS analysis, Annexin V-FITC/PI double-positive cells increased 48 h after treatment with TanIIA in a concentration-dependent manner (Figure 4). The apoptosis rates in EC-1 and ECa-109 cells treated with 1.3 *μ*g/mL of TanIIA were (40.21 ± 2.64)% and (60.62 ± 3.81)%, respectively, which was significantly higher compared with the control group [(5.68 ± 0.42) % and (5.08 ± 1.06) %, resp.] (*P* < 0.05).

### 3.3. TanIIA Induces Apoptosis through the Akt1/Bax/Bcl-2/Caspase-9/Caspase-3 Pathway and Induces S and G2/M Cell Arrest through the p53/p21/CDC2 and Cyclin B1 Complex Pathway

**Figure 6:**
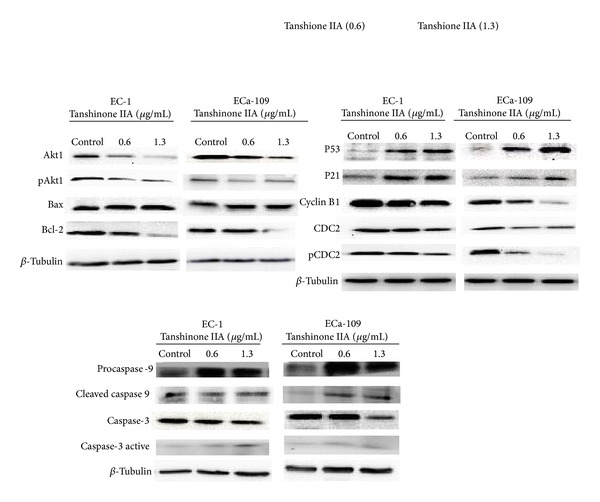
TanIIA induced esophageal cancer cells apoptosis through Akt/Bax/Bcl-2/caspase-9/caspase-3 pathway and arrested cell cycle in S and G2/M phase through p53/p21/CDC2 and cyclin B1 complex pathway. Total cell lysates were prepared and quantified by BCA protein assay kit. Western blots were detected with antibodies against Akt1, phospho-Akt1, p53, Bax, Bcl-2, procaspase-9, cleaved-caspase-9, caspase-3, caspase-3 active, p21, CDC2, phospho-CDC2, cyclin B1, and beta-tubulin. A representative blot is shown by independent experiments in triplicate.

We used Western blotting analysis to understand the mechanism through which TanIIA induces apoptosis and arrests the cell cycle in the S and G2/M phase. We detected the mitochondria-dependent signaling apoptotic pathway after TanIIA treatment for 48 h in EC-1 and ECa-109 cell lines and found that the expression of Akt1 and its phosphorylation proteins were inhibited, and an increased expression of Bax/Bcl-2 ratio (Figure 6). Bax induced mitochondrial damage and activated caspase-9 to form a holoenzyme complex (Figure 6). Caspase-3 was activated by the complex and then cleaved many key proteins in apoptosis (Figure 6).

TanIIA also increased p53 protein levels, which controlled the expression of p21 in the cell cycle (Figure 6). p21 interacted with DNA polymerase accessory factors to decrease the expression of CDC2 and inhibit its phosphorylation, preventing CDC2/cyclin B1 complex formation (Figure 6). Our results show that TanIIA treatment increases the protein expression of p53 and p21 and decreases the expression of CDC2, p-CDC2, and cyclin B1.

## 4. Discussion

Tanshinone IIA (TanIIA) is a traditional Chinese medicine confirmed to have antioxidant and anti-inflammatory effects in cardiovascular disease. It has also been reported to have inhibitory effects on many cancer cells* in vitro*. In the present study, we found that TanIIA inhibits cell proliferation and colony formation in EC-1 and ECa-109 cells in a time-dependent and concentration-dependent manner. TanIIA also induces cell apoptosis through mitochondria-dependent apoptotic signaling pathways and arrests the cell cycle in S phase and G2/M phase.

**Figure 7:**
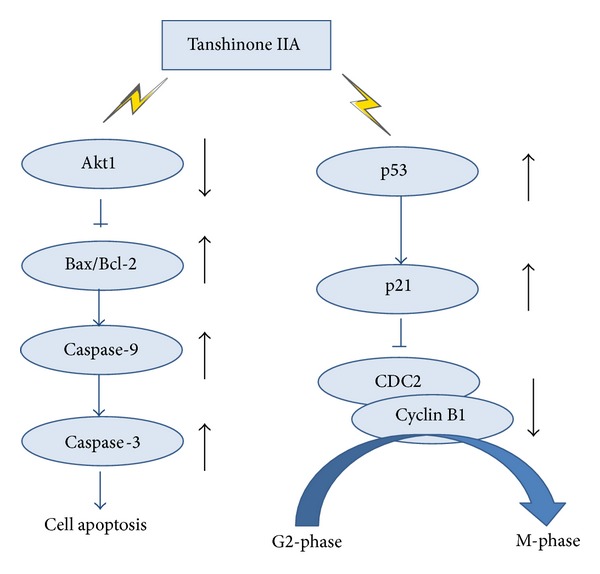
Model of the molecular mechanisms of TanIIA induced apoptosis and arrested cell cycle. TanIIA inhibits Akt and its phosphorylation, and this in turn increases the expression of Bax/Bcl ratio level, through mitochondrial damage to induce the activity of caspase-9. Caspase-3 is activated by this complex and results in apoptosis. Meanwhile, TanIIA activates p53, and this in turn increases the expression of p21. p21 interacts with some DNA polymerase accessory factors to decrease the expression of CDC2 and inhibit its phosphorylation, resulting in preventing CDC2/cyclin B1 complex formation.

TanIIA affects cell cycle distribution differently depending upon the tumor cell type and may have a complex mechanism in tumor cells [10]. In previous studies, two types of cell cycle distribution were reported. In one type, TanIIA partially arrested the cell cycle in the G0/G1 phase in tumor cells, including gastric cancer cells, breast cancer cells, and human glioma cells [11–14]. In the second type, tumor cells, such as human cervical cells and prostate cancer cells, were arrested in the S phase and/or G2/M phase [15–17]. In the present study, we found that TanIIA arrests the cell cycle in the S phase and G2/M phase in EC-1 and ECa-109 cells in a concentration-dependent manner at 48 h. Western blotting analysis showed that TanIIA increased p53 protein and p21 protein levels. Meanwhile, TanIIA downregulated CDC2 protein levels and cyclin B1 protein levels and inhibited CDC2 phosphorylation at Py15. p53 acts as a tumor suppressor that leads to the transcription of genes triggering cell cycle arrest at multiple sites* in vivo* [18]. Furthermore, p53 in human hepG2 cells and ovarian cancer cells arrests the cell cycle in the G2/M phase [19, 20]. P21 (named CDKN1A or WAF1) is an important intermediate through which p53 mediates its role as an inhibitor of cellular proliferation in response to DNA damage; its overexpression plays a critical role in cell cycle arrest [21, 22]. CDC2 is a member of the Ser/Thr protein kinase family. The CDC2 kinase regulates cell cycle progression at the S phase and G2/M phase in conjunction with cyclin B1 to form the CDC2-cyclin B1 complex, also known as maturation promoting factor (MPF) [19, 23, 24]. The activation of MPF is controlled through the dephosphorylation of CDC2 at tyrosine15 [25]. TanIIA decreased the phosphorylation of CDC2 at tyrosine15; then p-CDC2(pY15) decreases the expression of CDC2-cyclin B1 complex and arrests cell cycle in S phase and G2/M phase. Future investigations are needed to explore the mechanism through which TanIIA downregulates CDC2 levels. Based on our present study, we hypothesize a relationship with the expression of p21. Therefore, TanIIA may induce S and G2/M cell arrest in EC-1 and ECa-109 cells through the p53/p21/CDC2 and cyclin B1-complex signaling pathway (Figure 7).

In the present study, from the results of Hoechst 33258 staining and Annexin V-FITC/PI double staining, we found that TanIIA obviously induced apoptosis in EC-1 and ECa-109 cells at 48 h in a concentration-dependent manner. As the mitochondria-dependent apoptotic signaling pathway is a classical pathway involved, we investigated the expression levels of Akt1, Bcl-2, Bax, caspase-9, and caspase-3 activation through Western blotting analysis. The results showed that TanIIA decreased the expression of Akt1 and inhibited Akt1 phosphorylation at pS473. Akt1 is a Ser/Thr protein kinase with a key role in cell apoptosis, glycogen synthesis, and cell growth. It inhibits apoptosis by phosphorylating and inactivating several targets, including caspase-9 [26]. Thus, TanIIA potentially induces apoptosis by inhibiting the phosphorylation of Akt1.

Akt1 also inhibits apoptosis proteins of the Bcl-2 family, like Bax. Bcl-2 is an antiapoptotic protein that resides in the outer mitochondrial wall. In contrast, Bax is a proapoptotic molecule that promotes apoptosis through binding Bcl-2 and inhibiting the antiapoptotic function of Bcl-2 [27–30]. TanIIA treatment in esophageal carcinoma cells increased the expression of Bax protein levels. It also decreased the expression of Bcl-2 protein levels, and the change in Bax/Bcl-2 ratios indicated the effect of TanIIA in apoptosis.

Most drug compounds reportedly induce apoptosis through intrinsic death signaling pathways. The Bcl-2 family participates in this process and caspase-9 also plays a key role in this pathway [31]. The expression of procaspase-9 and cleaved caspase-9 increased in our study. The activation of caspase-9 is regarded as the earliest event in the caspase activation cascade [32]. Caspase-3 is a member of the apoptosis-execution functional group of caspases and cleaves many key proteins in apoptosis [7, 33, 34]. We found that TanIIA can increase the activation of caspase-3 and induce apoptosis. This indicates that TanIIA may act through the Akt/Bax/Bcl-2/caspase-9/caspase-3 signaling pathway to induce apoptosis in EC-1 and ECa-109 cells (Figure 7).

In conclusion, our results demonstrate that TanIIA can inhibit cell proliferation, arrest the cell cycle, and induce apoptosis of esophageal carcinoma cells. TanIIA induced apoptosis may be through the Akt1/Bax/Bcl-2/caspase-9/caspase-3 pathway and induced S and G2/M cell arrest may be through the p53/p21/CDC2 and cyclin B1 complex signaling pathway. Therefore TanIIA may serve as a potential anticancer drug in the treatment of esophageal cancer.
